# Spectrum of Thyroid Abnormalities among Children Living with HIV in Lagos, Nigeria

**DOI:** 10.1155/2019/1096739

**Published:** 2019-03-21

**Authors:** Adeseye Akinsete, Elizabeth Oyenusi, Babatunde Odugbemi, Tinuola Odugbemi, Edamisan Temiye

**Affiliations:** ^1^Department of Pediatrics, College of Medicine, University of Lagos/Lagos University Teaching Hospital, Nigeria; ^2^Department of Pediatrics, College of Medicine, University of Lagos/Lagos University Teaching Hospital/Pediatric Endocrinology Training Center for West Africa, Nigeria; ^3^Lagos State University Teaching Hospital, Nigeria; ^4^College of Medicine, University of Lagos, Nigeria

## Abstract

Thyroid disorders have been described in an adult population but are underreported in the pediatric population. The aim of this study was to determine the prevalence and describe the spectrum of thyroid abnormalities among HIV infected children on Highly Active Antiretroviral Therapy (HAART) in Lagos, Nigeria. This was a cross-sectional study carried out at a teaching hospital with an antiretroviral therapy (ART) center. Serum levels of thyroid stimulating hormone (TSH), free triiodothyronine (fT3), and free thyroxine (fT4) were analyzed in 83 children living with HIV on HAART and 51 controls. The prevalence of thyroid dysfunction and correlation of fT3, fT4, and TSH with duration on HAART, age, CD4 count, and nutritional status were assessed. Thyroid abnormalities were seen in 9.6% of the children living with HIV comprising subclinical hypothyroidism in 6%, euthyroid sick syndrome in 2.4%, and overt hypothyroidism in 1.2% as compared to 2% subclinical thyroid disease among the controls (p= 0.15). Hypothyroidism was correlated with CD4 count and viral load. None of the patients had clinical features of thyroid disease. Thyroid abnormalities were more prevalent among children living with HIV and yearly screening with follow-up is advocated.

## 1. Introduction

*Spectrum of Thyroid Abnormalities among Children Living with HIV in Lagos, Nigeria. *The Human Immunodeficiency Virus (HIV) and Acquired Immune Deficiency Syndrome (AIDS) are one of the commonest causes of childhood morbidity and mortality affecting an estimated two million children worldwide. Sixty-seven percent of these affected children reside in sub-Saharan Africa [1]. As at 2015, an estimated number of 3.5 million people were living with HIV/AIDS in Nigeria, (prevalence rate of 3.1%) with an estimated 260,000 children [2]. The initial approach to care of affected individuals was palliative [3] but, with the use of combination chemotherapy, there has been an improvement in both immunologic and clinical response with more individuals living for prolonged periods. Consequently, HIV/AIDS has become a chronic condition requiring life-long management [4–7].

The thyroid gland is an important regulator of cellular metabolism and growth, and dysfunction of the gland has been implicated in suboptimal functions of organs and systems within the body. The spectrum of thyroid dysfunction has been documented but mainly with regard to populations with HIV/AIDS outside sub-Saharan Africa (SSA) [8–10]. The prevalence of subclinical hypothyroidism among individuals living with HIV/AIDS has been documented to be higher than in the general population and highest among patients on HAART [11, 12]. Furthermore, the prevalence of Graves' disease has been reported to be higher among patients with immune reconstitution syndrome [13]. Nonthyroidal illness (sick euthyroid) has also been noticed to be quite common.in advanced AIDS [14–16], Finally, overt hypothyroidism has been reported in an estimated 2.6% of the HIV/AIDS population and this worsens the overall outcome of the affected individual [11, 12].

Thus, the aim of this current research was to determine the prevalence of thyroid dysfunction among children living with HIV and to identify the factors that may be associated with thyroid disorders among children living with HIV.

## 2. Experimental Design and Methods

### 2.1. Study Design and Ethical Considerations

This cross-sectional study was conducted at the AIDS Prevention Initiative (APIN) Clinic of the Lagos University Teaching Hospital, Lagos, Nigeria, from January 2017 to December 2017. Ethical approval was obtained from the Hospital's Health, Research and Ethics Committee before the commencement of the study. Informed consent was obtained from the parents/care-givers of the participants. In addition, those older than 7 years assented to the study. All children with documentary evidence of HIV older than 6 months were consecutively enrolled. The control participants were children who had documentary evidence of being negative for HIV and were attending routine clinics. Eighty-three (83) participants who were HIV positive and 51 who were HIV negative met the inclusion criteria. Known cases of chronic renal failure, thyroid disorder, treatment for thyroid dysfunction, and Hepatitis B/C positivity and those taking rifampicin were excluded from the study.

### 2.2. Enrolment of Participants

Following enrolment, the study participants and care givers were interviewed and demographic and clinical data were extracted from the patient's case notes (stage of disease, CD4 count/percentage (counts within the last one month to enrolment), ARVs, duration on HAART, nutritional status, Hepatitis B status, and HIV status). A detailed clinical examination including anthropometry with emphasis on the thyroid gland and cardiovascular system was conducted and recorded.

Serum thyroid stimulating hormone (TSH), free triiodothyronine (fT3), and free thyroxine (fT4) were analyzed using a Cobas 6000 analyzer by Roche Diagnostics USA following the manufacturer's guidelines.

### 2.3. Definition of Terms

The children were grouped and thus

1. Elevated serum TSH with corresponding low levels of T3 and T4 was overt hypothyroidism
2. Elevated serum TSH and normal thyroxine levels was subclinical hypothyroidism
3. Low serums T3 and T4 and low or normal TSH levels were sick euthyroid syndrome
4. Reduced serum TSH with corresponding high levels of T3 and T4 was overt hyperthyroidism
5. Reduced serum TSH and normal thyroxine levels was subclinical hyperthyroidism

### 2.4. Statistical Analysis

Data was input and analyzed using the Statistical Package for Social Sciences version 20 (IBM Corp). Univariate analysis was done for the variables of interest. Student's t test and Pearson's' correlation coefficient were used to test for associations between continuous variables while Chi-Square test was used to test for the association between categorical variables. A p value of < 0.05 was considered as statistically significant.

## 3. Results

### 3.1. General Characteristics

A total of 83 children living with HIV aged between 6 months and 18 years were enrolled in the study. The mean age was 9.23± 4.06 years with a male to female ratio of 2:1.  Table 1 shows the sociodemographic characteristics of the children living with HIV. Over 50% of the children were older than 5 years. Majority of the children were from the lower socioeconomic class. Most of the caregivers sampled had received at least 11 years of formal education.

### 3.2. Clinical Characteristics of Children Living with HIV

The clinical characteristics of the children living with HIV are shown in Table 2. Majority of the children had been living with the infection for greater than 5 years. While one-third had lived with the infection for less than five years, over two-thirds had been living with the virus for 6 years and beyond. Seventy percent had also been on drugs for greater than 6 years. Over two-thirds had good immunologic status from their CD counts results and viral load results.

### 3.3. Thyroid Function of Study Subjects

Serum thyroid function tests values were compared among the children living with HIV and controls. The prevalence of thyroid abnormality was 9.6% in the children living with HIV and 2% among the controls. (P=0.15). Hypothyroidism was the most common thyroid abnormality observed.

### 3.4. Association between Thyroid Function and HAART Type

We studied the effect of the different HAART regime on thyroid function and discovered that there was no association between the type of HAART used and the thyroid abnormalities.

### 3.5. Correlation Results of Variables

FT3 levels were correlated with CD4 count levels and viral load. There was no correlation between length of HAART use and thyroid function as well as age and nutritional status.

## 4. Discussion

The mean age of participants in this study was 9 years and most of the participants in this study were older than 5 years with more boys than girls. This reflects the strides achieved in the treatment of children living with HIV thus converting an otherwise fatal disease to a chronic disease [4–7]. Approximately one-third of the participants were in their second decade of life and they had been living with the disease for that length of time. This is because the most prevalent method of transmission is from mother to child. There have been challenges with the uptake of HAART in certain settings; however, this was not the case with these groups of participants. Majority of them had been on medications for over five years and an indirect measure of adherence to medications is the immunologic state as well as the state of virologic suppression of the individual [17, 18].

The thyroid gland is an important organ in growth with different mechanisms postulated for disease states among individuals living with HIV/AIDS [9–11]. None of the participants in this cohort had an enlarged thyroid gland. The virus and even certain medications have been implicated as the cause of thyroid abnormalities among several cohorts [13–15]. Among this study population, thyroid abnormalities were clinically more apparent among the population of children living with HIV. Most of the children had sub clinical hypothyroidism which has been described as the commonest form of thyroid abnormality among children living with HIV [19, 20] (Table 3). The clinical significance of this condition remains unknown but a progressive increase in thyroid binding globulin has been implicated in the condition [21]. It is important to note that clinical manifestation of hypothyroidism is rarely seen among children living with HIV [22]. Those with subclinical hypothyroidism usually have high TSH levels, which has been associated with more progressive disease [22]. However, the cross-sectional nature of this study did not allow for assessing progressive disease.

Euthyroid sick syndrome was another thyroid abnormality seen in the children living with HIV in this study (Table 3). It has been described as an energy conserving adaptive mechanism during stressful periods [22]. In an earlier study among Thai children [20], the prevalence was 14% and the condition was associated with low CD4 counts. Since it is a protective phenomenon, expectedly, the prevalence was lower among this cohort reflecting their favorable response to treatment. In this study, the prevalence of sick euthyroid syndrome was 2.4%; however, it was not associated with reduced CD4 count. Approximately two-thirds of the study population had CD4 count greater than 500 as well as reduced viral loads (Table 2). The participants with sick euthyroid syndrome in this cohort did not have clinically evident thyroid disease like in the earlier report [20]. This may be related to the easy accessibility of medications and support from the dedicated service for children living with HIV.

The rate of overt hypothyroidism in this study was 1.2% which was far lower than the 10% and 11% reported by two earlier studies in India [23, 24]. Furthermore, one of the earlier studies had participants with more advanced disease states [23]. However, this was not the case in the study by Ranabir et al. [24] and the current study. Like the two earlier reports, this study did not have any participant with clinical signs of hypothyroidism. It must be pointed out that overt disease is said to be extremely rare in people living with HIV [22].

The use of HAART has been correlated with improved outcomes and survival; however, there have been suggestions of increased thyroid abnormalities among children who were on HAART [25, 26]. The prevalence of thyroid abnormalities was reported to be significantly increased with further exposure to HAART [26]. In a retrospective study in Germany, subclinical disease was seen in 6% of the studied population and it correlated with duration of HAART use [27]. However, in this study, there was no correlation between length of HAART use and thyroid abnormalities. This finding was corroborated by Ranabir et al. [24] who reported similar prevalence rates among children living with HIV irrespective of HAART use.

**Figure 1:**
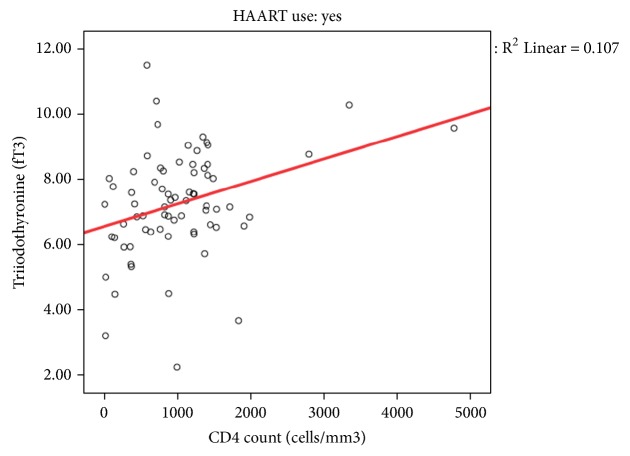
Thyroid hormone level and CD4 count.

**Figure 2:**
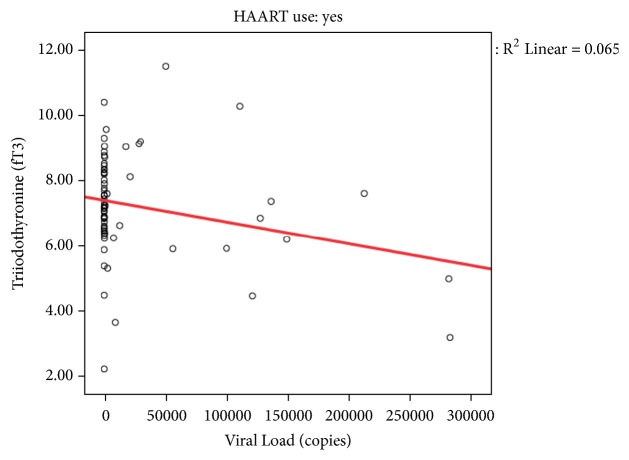
Thyroid hormone level and viral load.

The index study showed that thyroid dysfunction correlated with CD4 count as well as with viral load. It had been reported that low T3 levels correlated with severe immunosuppression [20] (Figures 1 and 2). This finding corroborated what was described earlier among Thai children where those with moderate to severe immunosuppression had thyroid dysfunctions [20]. It is believed that thyroid response and function are impaired in severe immunosuppression by an increase in the concentration of circulating thyroid binding globulin [22]. Several other reasons have been adduced for the increased prevalence of thyroid hypo functioning among children living with HIV especially among those with moderate to severe immunosuppression and they include possible drug reactions, recurrent infections, disease progression, or the interaction of these factors in a child exposed to multiple insults [19, 28, 29].

Many cases of thyroid dysfunction in individuals with HIV have been attributed to HAART with hypothyroidism attributed to protease inhibitors and hyperthyroidism with nonnucleoside reverse transcriptase inhibitors [30]. However, in this study, there was no association between specific HAART regimes and thyroid dysfunction (Table 4) and this was corroborated by Nelson et al. [30].

In conclusion, this study highlights that thyroid abnormalities occur in children living with HIV and it may be important to perform yearly screening especially among children with moderate to severe immunosuppression.

## Figures and Tables

**Table 1:** Characteristics of children living with HIV.

	HIV positive children
Characteristic	Frequency (%)
*Sex*	
Male	55 (66.3)
Female	28 (33.7)
*Age Range*	
< 1year	1 (1.2)
1-5years	14 (16.9)
6-10years	37 (44.6)
>10years	31 (37.3)
*Educational status of caregiver*	
Primary	11 (13.3)
Secondary	41 (49.4)
Tertiary	23 (27.7)
No formal education	8 (9.6)
*Socioeconomic status of caregiver*	
High	2 (2.4)
Middle	28 (33.7)
Low	53 (63.9)

**Table 2:** Clinical characteristics of Children living with HIV.

Variable	Frequency (%)
*HIV history*	
Mean years living with HIV	
0-5years	24 (28.9)
6-10years	38 (45.8)
>10years	21 (25.3)
*Duration on HAART*	
<5years	25 (30.1)
5-10years	11 (13.3)
10years	47 (56.6)
*CD4 Count*	
<200	20 (24.1)
200-500	9 (10.8)
>500-1000	21 (25.3)
>1000	33 (39.8)
*Viral load*	
<1000copies	62 (74.7)
1000-5000copies	3 (3.6)
>5000-10000copies	2 (2.4)
>10000copies	16 (19.3)

**Table 3:** Thyroid function values for the study participants.

	HIV positive children (n=83)	HIV negative children (n=51)	Fisher's exact p-value	
	Frequency (%)	Mean (SD)		
*Thyroid Function*				
Normal	75 (90.4)	50 (98.0)	0.15	
Abnormal	8 (9.6)	1 (2.0)		
*Specific thyroid abnormalities*				
Subclinical	5 (6.0)	1 (98.0)		
Sick euthyroid	2 (2.4)	0 (0.0)		
Overt thyroid disease	1 (1.2)	0 (0.0)		

Thyroid hormone levels	HIV positive children (n=83)	HIV negative children (n=51)	Mean difference (P-value)	Normal range
	Mean ± (SD)	Mean (SD)		

T3	7.2 ± 1.5	5.74 ± 1.3	1.46** (0.00) ∗∗**	3.10-6.80
T4	15.8 ± 2.7	18.46 ± 3.6	-2.67** (0.00) ∗∗**	12.00-22.00
TSH	3.2 ± 2.8	2.58 ± 1.4	0.63 (0.08)	0.27-4.20

*∗∗*P<0.01

**Table 4:** Association between thyroid function and HAART type.

HAART Combination	Normal	Abnormal	Chi square	p-value
AZT + 3TC + NVP	60 (89.6)	7 (10.4)	5.3	0.26
ABC + LPV/r + 3TC	2 (100.0)	0 (0.0)		
ABC + 3TC + EFV	1 (50.0)	1 (50.0)		
ABC+ 3TC + LPV/r	12 (100.0)	0 (0.0)		

AZT: Zidovudine, 3TC: Lamivudine, NVP: Nevirapine, ABC: Abacavir, LPV/r: Lopinavir, and EFV: Efavirenz

## Data Availability

The database is available with the corresponding author and can be sent to researchers who wish to validate the results.

## Additional Points

*Limitations*. The study was cross-sectional in nature and it was difficult to assess linear relationship between some of the variables.
